# Lactic acid bacteria from Ethiopian traditional beverage, *Tella*: technological and metabolic profiles for industrial application

**DOI:** 10.71150/jm.2409008

**Published:** 2025-01-24

**Authors:** Gashaw Assefa Yehuala, Jaein Choe, Nurelegne Tefera Shibeshi, Kumsa Delessa, Asnake Desalegn, Mi-Kyung Park

**Affiliations:** 1 School of Food Science and Biotechnology, and Food and Bio-Industry Research Institute, Kyungpook National University, Daegu 41566, Republic of Korea; 2 Biotechnology and Bioprocess Center of Excellence, Addis Ababa Science and Technology University, Addis Ababa P.O. Box 16417, Ethiopia; 3 School of Chemical and Bio-Engineering, Addis Ababa Institute of Technology, Addis Ababa University, Addis Ababa P.O. Box 385, Ethiopia; 4 Department of Microbial, Cellular and Molecular Biology, College of Natural and Computational Science, Addis Ababa University, Addis Ababa P.O. Box 1176, Ethiopia

**Keywords:** stress tolerance, acidification, metabolite profile, starter culture, *Tella*, LAB

## Abstract

*Tella* is a traditional beverage widely accepted by consumers, despite the lack of product consistency owing to its reliance on natural fermentation. This study aimed to identify potential industrial lactic acid bacteria (LAB) starter cultures based on their technological properties. Seven LAB strains isolated from *Tella* were characterized for their carbohydrate utilization, salt content, temperature, and acid tolerances, growth and acidification rates, and metabolite profiles. Most strains efficiently utilized various carbohydrates, with *Lactiplantibacillus plantarum* TDM41 showing exceptional versatility. The strains exhibited similar growth characteristics. Principal component analysis of stress tolerance properties revealed that *L. plantarum* TDM41, *Pediococcus pentosaceus* TAA01, and *Leuconostoc mesenteroides* TDB22 exhibited superior tolerance ability. Strong acidification properties were detected in the *L. plantarum* TDM41, *P. pentosaceus* TAA01, and *Leuconostoc mesenteroides* TDB22 strains after 24 h incubation at 30°C. *L. plantarum* TDM41 displayed the fastest acidification rate throughout the analysis period. All LAB strains produced significant amounts of diverse organic acids, including lactic acid, citric acid, acetic acid, malic acid, and succinic acid, with lactic acid being the primary acid produced by each strain. Overall, strains *L. plantarum* TDM41 and *P. pentosaceus* TAA01 prove to be potential candidates for *Tella* industrial starter cultures and similar cereal products owing to their robust technological properties.

## Introduction

*Tella*, a traditional Ethiopian beer, embodies centuries of cultural heritage and traditional craftsmanship. It is the most extensively brewed and consumed alcoholic beverage, particularly enjoyed during social occasions, such as the hosting of guests, holidays, weddings, funerals, community-serving events (ekub), and agricultural activities (Fentie et al., 2020; Lemi, 2020). *Tella* is brewed from various cereals, including maize, barley, wheat, sorghum, millet, and teff, with grain selection varying according to local traditions (Andualem et al., 2017; Ashenafi, 2006). Although minor changes occur during processing across different localities, the fundamental preparation steps, that is, the production of “tejet,” “tenses,” and “difdif,” are consistent countrywide (Fentie et al., 2020; Yehuala et al., 2024).

*Tella* is rich in minerals, flavonoids, and polyphenolic compounds, providing multiple biological benefits, such as protection against free radicals, cancer, and aging (Birhanu et al., 2021; Shewakena et al., 2017). Despite its significant nutritional contribution and cultural prominence, *Tella*’s production still relies on indigenous and spontaneous fermentation methods, resulting in considerable variability in its quality, safety, and consistency (Holzapfel, 2002; Hotessa and Robe, 2020; Lemi, 2020). Spontaneous cereal-based alcoholic fermentations typically involve an association between lactic acid bacteria (LAB) and yeast, as yeast growth is favored by acidification (Puerari et al., 2015). Traditional *Tella* fermentation takes approximately 2 weeks and can occasionally lead to uncertainties in achieving the desired product quality and safety (Berhanu, 2014; Tekle et al., 2019).

Considering the need for standardized *Tella* production and quality control, developing defined starter cultures for its fermentation is urgently required. Controlled fermentation is more efficient in ensuring consistent product quality during large-scale production (Carnevali et al., 2007). The fermenting microorganisms in *Tella* include *Saccharomyces* spp., *Lactobacillus* spp., *Acetobacter* spp., and *Bacillus* spp. (Hotessa and Robe, 2020; Lemi, 2020; Samuel and Berhanu, 1991; Tekle et al., 2019;). LAB, the focus of the present study, are crucial in cereal fermentation processes. They convert carbohydrates into organic acids, particularly lactic acid, which imparts desirable flavors, textures, and preservative properties to fermented products (Achi and Ukwuru, 2015). Moreover, LAB enhance the nutritional value of cereals by synthesizing vitamins (groups B and K), folate, the amino acid lysine, and micronutrients (Tamene et al., 2019; Tangyu et al., 2019).

To achieve controlled *Tella* fermentation, inoculation with defined starter cultures, especially autochthonous ones, adapted to the fermentation conditions and that contribute to a unique flavor, aroma, and other characteristics is required (Di Cagno et al., 2013). Using these indigenous cultures helps preserve the beverage’s traditional and authentic qualities. Therefore, selecting LAB strains with desirable technological characteristics from the spontaneous fermentation of the matrix of interest, *Tella* in this case, is an excellent means of obtaining useful autochthonous cultures for commercial purposes (Sáez et al., 2017). The key technological features for the selection of LAB starters include LAB’s carbohydrate utilization potential, growth and acidification rates, stress tolerance, metabolite profiles, and compatibility with other strains (Coda et al., 2010; Manini et al., 2016; Sáez et al., 2017; Sawadogo‐Lingani et al., 2008; Speranza et al., 2017).

In our previous work (Yehuala et al., 2024), seven LAB strains, including *Pediococcus pentosaceus* TAA01, *Latilactobacillus curvatus* TAA04, *Leuconostoc mesenteroides* TDB19, *Latilactobacillus curvatus* TDB21, *Leuconostoc mesenteroides* TDB22, *Latilactobacillus curvatus* TDM40, and *Lactiplantibacillus plantarum* TDM41, were isolated from *Tella*. Their probiotic properties and alcohol tolerance abilities were explored to select potential functional autochthonous starters. Notably, *P. pentosaceus* TAA01, *L. mesenteroides* TDB22, and *L. plantarum* TDM41 were found to exhibit promising functional properties, including a low pH, bile salt and alcohol resistance, antimicrobial activity, and auto-aggregation and coaggregation properties. However, evaluating the technological and organoleptic performance of these isolated strains remains necessary to facilitate their implementation as industrial starter cultures for beverage fermentation. Therefore, the present study aimed to evaluate the carbohydrate utilization potential, growth characteristics, stress tolerance properties, acidification rates, and metabolite profiles of LAB strains for subsequent use in co-culture with yeast cells for *Tella* fermentation.

## Materials and Methods

### LAB and growth conditions

*P. pentosaceus* TAA01, *L. curvatus* TAA04, *L. mesenteroides* TDB19, *L. curvatus* TDB21, *L. mesenteroides* TDB22, *L. curvatus* TDM40, and *L. plantarum* TDM41 were used in this study. Each strain was cultured in de Man–Rogosa–Sharpe (MRS; Kisan Bio Co., Ltd., Korea) broth under anaerobic conditions at 30°C for 48 h, prior to use.

### Carbohydrate utilization potential

Each LAB strain’s ability to utilize different sugars, such as D (+)−glucose, maltose, D−fructose, sucrose, D (+)−galactose, and D−sorbitol, was assessed following the method outlined by Hedberg et al. (2008), with minor modifications. Each sugar was dissolved in distilled water to produce a 2% solution, which was subsequently sterilized using a 0.22-µm syringe-driven filter unit (GVS Filter Technology Co., Ltd., USA). These sugar solutions were subsequently mixed in equal volumes with peptone broth (Difco, Maryland, USA) to prepare the sugar media. Thereafter, 200-μl cell suspensions from twice-washed overnight cultures of each LAB strain were separately inoculated into 4.5 ml of sugar medium (pH 6.75). All cultures were subsequently incubated at 30°C for 48 h. The pH of each sample was measured at the beginning and after 48 h of incubation, with uninoculated media serving as a control.

### Growth characteristics

Each LAB strain’s growth characteristics were investigated using a method adapted from Üçok and Sert (2020), with certain modifications. Briefly, each LAB strain’s overnight culture was inoculated into MRS broth at a concentration of 1% (10^6^ CFU/ml). Uninoculated MRS broth served as a control. Each LAB strain’s growth was monitored by measuring its optical density at 600 nm (OD_600_) every hour at a temperature of 30°C using a Synergy™ H1 microplate reader (Agilent Technologies Inc., USA). From the generated growth curves, the lag phase was identified as the initial period during incubation when no significant change in OD occurred, followed by the exponential phase, characterized by a rapid increase in OD. Finally, the end of the exponential phase was marked by the point at which OD no longer exhibited a significant increase. To calculate the specific growth rate, data points between the 4^th^ and 10^th^ h of incubation were selected, capturing the exponential growth phase common to all LAB strains. These data were subsequently subjected to exponential trendline analysis in Microsoft Excel, generating equations in the following form:


$$Y\text{­}=\text{­}Y_{O}\times e^{\mu t}$$

Where Y represents the OD_600_ at time t, Y_0_ is the initial OD_600_ at the initiation of exponential growth, μ denotes the specific growth rate, and t signifies time. The goodness of fit for each trendline was evaluated using the R-squared (R^2^) value provided by Excel to ensure the reliability of the fitted model. Additionally, the doubling time (Td) was calculated using the formula:


Td=In2/μ

### Stress tolerance properties

Each LAB strain’s stress tolerance was determined by assessing its ability to withstand the challenges posed by salt, temperature, and acidic pH. The assessments were conducted following established methodologies, as described in previous studies (Bevilacqua et al., 2010; Speranza et al., 2017). For salt tolerance, NaCl concentrations ranging from 2% to 10% were added to MRS broth. pH levels were adjusted to 3.5, 4.0, and 4.5 using 1 N HCl in separate batches of MRS broth to assess acidic pH tolerance. Temperature tolerance was evaluated at 15, 37, and 45°C. Each LAB strain was inoculated at 10^6^ CFU/ml under each condition. Microbial growth was monitored by measuring OD_600_ after 48 h using a microplate reader (Multiskan™ GO; Thermo Fischer Scientific, Finland). Control samples of unmodified MRS broth inoculated with each Lab strain were incubated at 30°C for comparison. The data for each case are expressed as a growth index (GI), which is a relative measure comparing growth under experimental conditions to that of the control. The GI was calculated using the following formula:


(3) $$GI=(A_{S}/A_{C})\times 100$$

Where A_S_ represents the absorbance of the samples under different conditions, and A_C_ denotes the absorbance of the control.

### Acidification kinetics

The strains’ acidification kinetics were evaluated in a sterile *Tella* substrate extract (STSE) broth according to the method proposed by Sáez et al. (2017), with certain modifications. *Tella* substrates, including gesho (*Rhamnus prinoides*), bikil, kita, and derekot, were prepared using traditional methods, as described by Berhanu (2014). Thereafter, substrates with the following composition (g): gesho, 12.5; bikil, 12.5; kita, 25; and derekot, 100 were suspended in 1 L of distilled water and stirred for 2 h. Each mixture was subsequently sterilized at 121°C for 15 min. After centrifugation at 8,000 × g and 4°C for 10 min, the supernatant was adjusted to pH 6.5 to evaluate the acidification ability of each LAB strain. Each LAB strain was grown in MRS broth for 18 h at 30°C. The cultures were centrifuged at 8,000 × g for 10 min, and the obtained pellet was washed twice with phosphate-buffered saline (PBS; Welgene Inc., Korea). Each culture was subsequently resuspended and adjusted to 8 log CFU/ml in PBS. These cell suspensions were inoculated (2%) into STSE and incubated at 30°C for 24 h. The pH of each sample was measured at 0, 3, 6, 9, and 24 h using a digital pH meter (OHAUS Corporation, USA).

### Organic acid production

The production of the most common organic acids, including acetic acid, lactic acid, citric acid, malic acid, and succinic acid, via STSE fermentation with each LAB strain was determined. Eight treatments were performed using different LAB strains: (T1) STSE without fermentation, (T2) STSE fermented with *P. pentosaceus* TAA01, (T3) STSE fermented with *L. curvatus* TAA04, (T4) STSE fermented with *L. mesenteroides* TDB19, (T5) STSE fermented with *L. curvatus* TDB21, (T6) STSE fermented with *L. mesenteroides* TDB22, (T7) STSE fermented with *L. curvatus* TDM40, and (T8) STSE fermented with *L. plantarum* TDM41. The LAB strains were cultured in MRS broth at 30°C for 18 h, centrifuged (8,000 × g, 10 min), washed twice with PBS, and adjusted to 8 log CFU/ml. The obtained cell suspensions were inoculated (2%) in STSE and incubated at 30°C for 24 h. The fermented samples were subsequently centrifuged at 8,000 × g and 4°C for 10 min and filtered through a 0.45-µm syringe filter (GVS Filter Technology Co., Ltd., USA). Organic acids were quantified using high-performance liquid chromatography (HPLC; Shimadzu Co., Japan) following a method adapted from Ferreira et al. (2022). HPLC analysis was conducted using an Agilent Hi-Plex H Ion-Exchange Column (300 × 7.7 mm), with the column oven temperature maintained at 65°C. Organic acids were detected using a Shimadzu RID-10A Refractive Index Detector (Shimadzu Corporation, Japan). Sulfuric acid (5 mM) was used as the eluent at a flow rate of 0.6 ml/min and sample volume of 20 µl. For each sample, standard solutions of lactic acid, citric acid, acetic acid, malic acid, and succinic acid were prepared in distilled water to generate calibration curves. The compounds were identified by comparing their retention times to those of the standards, and their concentrations were determined using the calibration curves.

### Statistical analysis

All experiments were conducted in triplicate, and the results are presented as the mean ± standard deviation. Data were analyzed using a one-way analysis of variance in GraphPad Prism 8.3.0 (GraphPad Software Inc., USA), followed by Tukey’s post hoc test for mean comparisons, with statistical significance set at P < 0.05. All figures, except for the principal component analysis (PCA) plots, were generated in GraphPad Prism. PCA was conducted using Minitab 19.2 Statistical Software (Minitab Inc., State College, USA) to evaluate stress tolerance properties, including salt, temperature, and acid tolerance, and to identify strains with superior resilience. PCA was also utilized to assess correlations between LAB strains and the organic acids produced.

## Results and Discussion

### Carbohydrate utilization potential of LAB strains

Fermentation is an ancient, simple, and cost-effective means of improving the shelf life, hygiene, nutritional value, and organoleptic quality of foods (Sáez et al., 2017). When selecting LAB for food fermentation, considering the various technological properties of LAB strains relevant to facilitating the fermentation process and enhancing final product quality, such as the carbohydrate utilization potential, is essential (Gänzle, 2014; Manini et al., 2016). The carbohydrate utilization potential of the LAB strains is presented in Table 1. The strains reduced the pH value of the sugar media, demonstrating the strains’ varied abilities to utilize different carbohydrates. Most strains efficiently utilized the sugars, including D(+)−glucose, maltose, D−fructose, sucrose, D(+)−galactose, and D−sorbitol. However, the strains’ utilization of sorbitol was generally low or absent, except that of *L. curvatus* TDB21 and *L. plantarum* TDM41. *P. pentosaceus* TAA01, *L. mesenteroides* TDB19, *L. mesenteroides* TDB22, and *L. curvatus* TDM40 did not utilize sorbitol at all. Notably, *L. plantarum* TDM41 displayed outstanding performance by exhibiting high utilization (+++) of all tested carbohydrates, including sorbitol at a low utilization level (+). This exceptional metabolic versatility underscores its suitability for diverse fermentation contexts, potentially enhancing product characteristics and broadening applications. The findings are strongly consistent with those reported by Rahmati (2017). *L. plantarum*’s high carbohydrate-utilization efficiency across various carbohydrates has also been reported in previous works (Divyashree et al., 2024; Rahmati, 2017).

Strain mixtures with different carbohydrate metabolisms are often used to ensure optimal acidification and sensory properties (Gobbetti, 1998). The findings underscore the importance of selecting appropriate LAB strains based on their metabolic capabilities to control fermentation processes and product outcomes.

### Growth characteristics

**Fig. 1.**
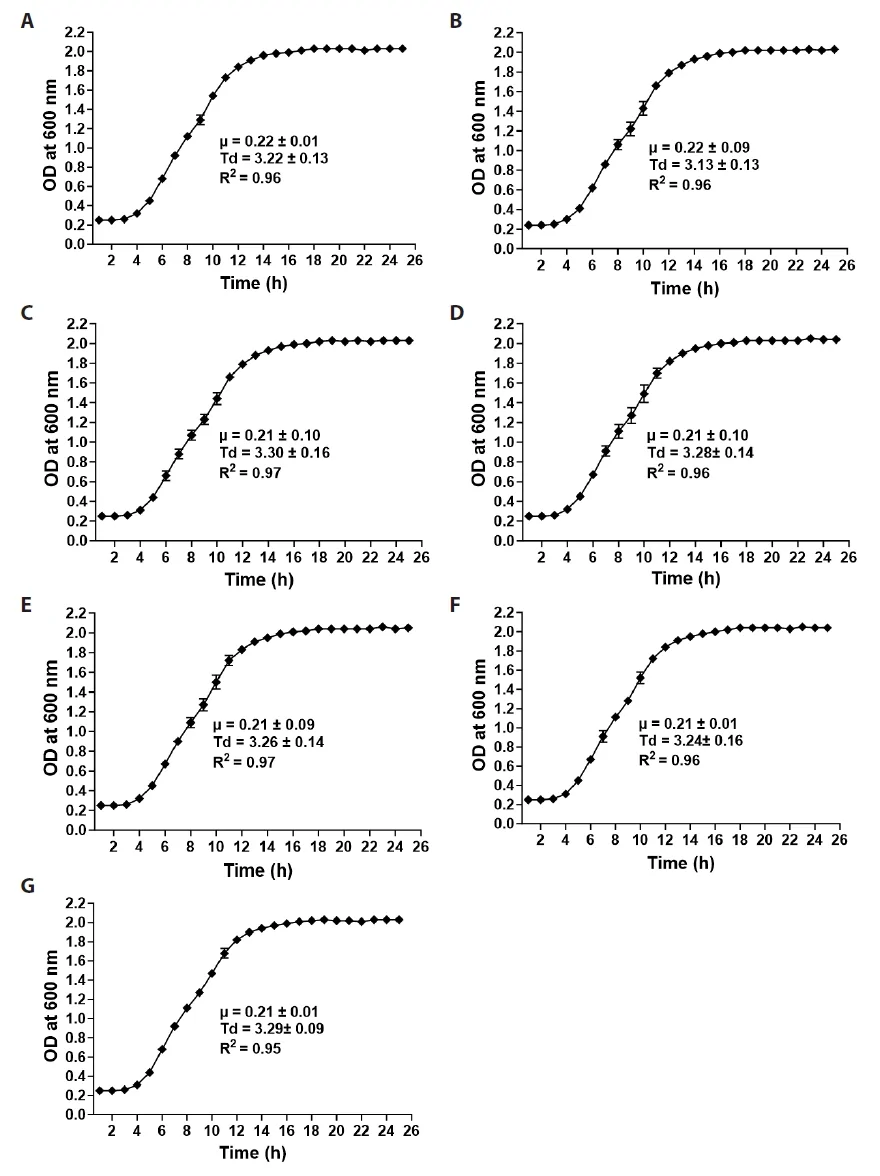
Growth curves and kinetic parameters of (A) *P. pentosaceus* TAA01, (B) *L. curvatus* TAA04, (C) *L. mesenteroides* TDB19, (D) *L. curvatus* TDB21, (E) *L. mesenteroides* TDB22, (F) *L. curvatus* TDM40, and (G) *L. plantarum* TDM41 incubated in MRS broth at 30°C for 24 h. Values are expressed as the mean ± SD (n=3).

The metabolic activity rate of the starter culture is an important factor to be considered when selecting strains starter cultures for starter culture development (Holzapfel, 2002). High growth rates can shorten fermentation times and improve the specific strain’s viability by inhibiting the growth of undesirable microorganisms in the raw material (Marklinder & Lönner, 1992). The growth curves and key growth kinetic parameters of the LAB strains are presented in Fig. 1. As shown in the figure, all strains exhibited a consistent lag phase of approximately 4 h, indicating a similar initial adaptation period. Most strains displayed an exponential phase duration of 11 h, except for *L. curvatus* TAA04, which exhibited a relatively shorter exponential phase of 10 h. Specific growth rates (µ) and Tds during the exponential growth phase (between the 4^th^ and 10^th^ h) were also calculated (Fig. 1). The average specific growth rate was 0.21 ± 0.00 h^–1^, and the average Td was 3.25 ± 0.06 h. No significant differences (P ≥ 0.05) in specific growth rates and Tds occurred among the strains. These findings are consistent with those obtained by Goswami et al. (2017), who reported similar growth characteristics among the different LAB strains. Additionally, the observed lag phases were shorter than those reported by Kang et al. (2020), indicating that the strains had better adaptation potential to the fermentation medium. To ensure their suitability for industrial applications, further large-scale studies will be needed to confirm whether the growth kinetics observed in this study remain consistent in larger fermentation volumes.

### Stress tolerance properties

Stress tolerance is a key criterion for selecting LAB strains as starter cultures in industrial fermentation. Effective LAB strains must withstand several stressors, including osmotic, acidic, and temperature fluctuations, which vary across substrates and fermentation stages (Vinicius De Melo Pereira et al., 2020). Initially, LAB encounter osmotic stress from solutes such as sugars or salt, depending on the substrate composition (Yousef & Courtney, 2003). During fermentation, LAB face additional challenges, including temperature fluctuations and acid buildup (Yousef & Courtney, 2003). Temperature increases and pH decreases are among the most common environmental stresses these starter cultures must withstand. In this study, LAB strains were evaluated for their tolerance to osmotic stress, temperature variations, and acidic conditions, all of which are essential for their suitability in industrial applications. The following sections detail each strain’s responses to these factors, relating to their potential for industrial applicability.

**Fig. 2.**
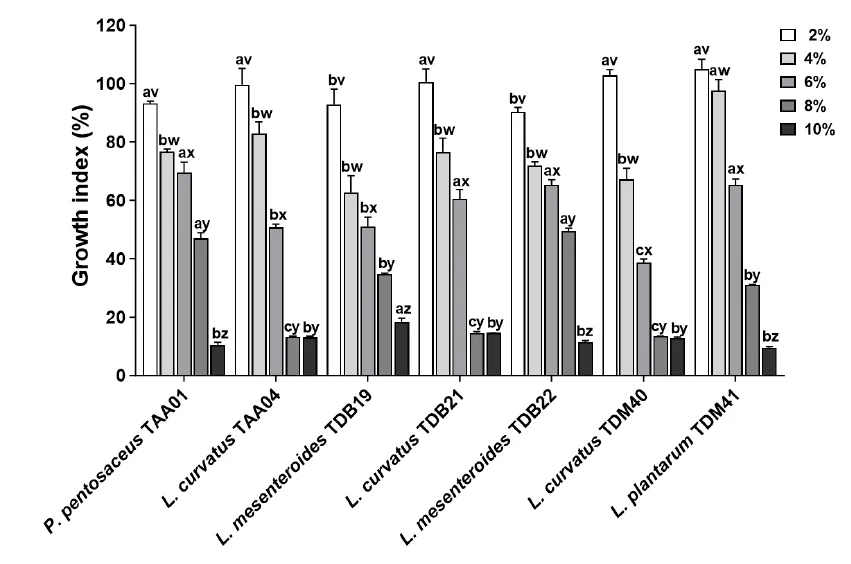
Stress tolerance abilities of LAB strains exposed to 2, 4, 6, 8, and 10% (w/v) salt during growth in MRS broth. Values are expressed as the mean ± SD (n=3). Different superscript letters (a–c) represent significant differences (P < 0.05) between different isolates under the same treatment as well as (v–z) those within the same isolate between different treatments.

**Salt tolerance:** The capacity to withstand osmotic stress is a critical factor when selecting native LAB strains for various industrial purposes, including *Tella* fermentation. At a 2% NaCl concentration, all strains displayed GIs ranging from 90.31 ± 1.52% to 104.95 ± 3.42%. Notably, *L. curvatus* TDB21, *L. curvatus* TDM40, and *L. plantarum* TDM41 exhibited enhanced growth in response to increased salt concentration, as illustrated in Fig. 2. At a 4% NaCl concentration, the LAB strains yielded an average GI value > 76.55 ± 11.44% (Fig. 2). Increasing the salt concentration to 6% exerted more influence on strain growth. Among the strains, *P. pentosaceus* TAA01, *L. mesenteroides* TDB22, and *L. plantarum* TDM41 displayed superior tolerance (GI > 65.28 ± 1.80%) at 6% NaCl. Growth inhibition on all LAB strains intensified, with average GI values of 29.07 ± 15.72% and 12.87 ± 2.95%, as the salt concentration was increased to 8% and 10%, respectively. These findings agree with those of a study by Speranza et al. (2017), who found a salt increase of 7.5% to affect most LAB growth, yielding GI values between 30% and 75%. At each salt concentration, significant growth-inhibition differences occurred between strains of either similar species, such as *L. mesenteroides* TDB19 and *L. mesenteroides* TDB22, or different species, such as *L. mesenteroides* TDB19 and *P. pentosaceus* TAA01. This indicates that tolerance to salt concentration may be both species- and strain-dependent (Sánchez García et al., 2007). The salt tolerance observed in these LAB strains provides valuable insights into their suitability for various industrial fermentations. All strains demonstrated high growth rates at salt concentrations (2–4% NaCl), making them suitable for controlled fermentations in mildly salted products, such as sourdough bread, fermented pizza, and some cheeses products (Hu et al., 2024). However, strains like *P. pentosaceus* TAA01, *L. mesenteroides* TDB22, and *L. plantarum* TDM41, which exhibit better tolerance at higher salt concentrations (6% NaCl and above), are particularly suited for high-salt fermentations in products requiring robust osmotic resilience, such as fermented meats and aquatic products (Hu et al., 2024; Speranza et al., 2017).

**Fig. 3.**
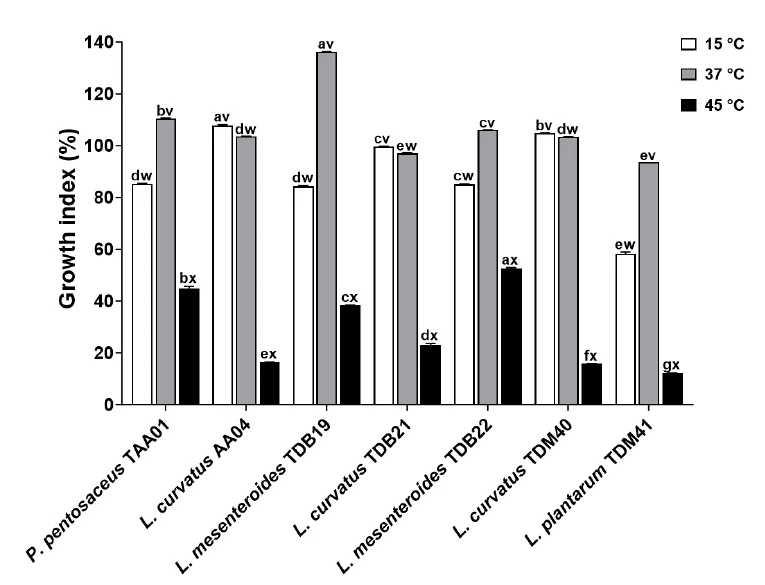
Stress tolerance abilities of MRS broth-grown LAB strains exposed to temperatures of 15, 37, and 45°C. Values are expressed as the mean ± SD (n=3). Different superscript letters (a–g) represent significant differences (P < 0.05) between different isolates under the same treatment, while (v–x) denote significant differences between different treatments within the same isolate.

**Fig. 4.**
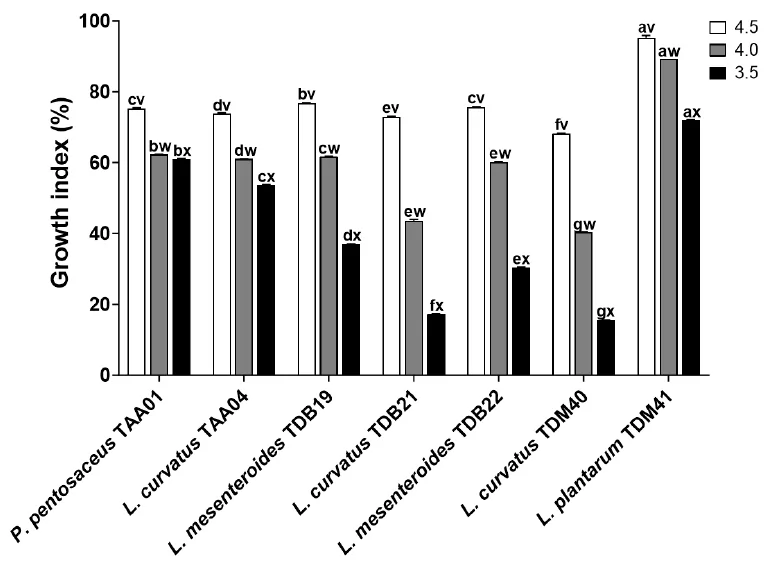
Stress tolerance abilities of MRS broth-grown LAB strains exposed to acidic conditions at pH 3.5, 4.0, and 4.5. Values are expressed as the mean ± SD (n=3). Different superscript letters (a–g) represent significant differences (P < 0.05) between different isolates under the same treatment, while (v–x) signify significant differences between different treatments within the same isolate.

**Temperature tolerance:** Another crucial factor to be scrutinized was temperature variation. The growth patterns of the LAB strains were meticulously analyzed at 15, 37, and 45°C (Fig. 3). The average GIs were 89.32 ± 16.90%, 107.20 ± 13.94%, and 28.99 ± 16.03% at 15, 37, and 45°C, respectively. Among these, 45°C treatment exerted the most profound impact on the GI of each strain. All strains yielded GI values ranging from 93.71 ± 0.05% to 110.56 ± 0.20% at 37°C. The highest GIs at 37°C indicate that these strains may also possess probiotic qualities (Kathade et al., 2020). At a 15°C incubation temperature, *L. curvatus* TAA04 exhibited the maximum GI (107.85 ± 0.20%), whereas *L. plantarum* TDM41 yielded the minimum GI (58.26 ± 0.61%). Notably, GIs exceeding 100% were displayed by the *L. curvatus* TAA04 and *L. curvatus* TDM40 LAB strains, particularly at the 15°C incubation temperature. This high GI may be attributed to the extended incubation period of 48 h, which potentially caused LAB strain autolysis among the controlled samples after they had reached the stationary phase (Kang et al., 1998). In turn, autolysis possibly led to a decrease in the OD values of the control samples, causing the GIs of the mentioned strains to surpass 100% at 15°C (Kang et al., 1998). Concerning growth patterns at 45°C, *L. mesenteroides* TDB22 yielded the highest GI (52.42 ± 1.17%), whereas *L. plantarum* TDM41 generated the lowest (12.21%). Generally, under 48 h incubation, all strains exhibited better GI values at low (15°C) than at high (45°C) temperatures. Overall, at 15°C, the three *L. curvatus* strains, namely, *L. curvatus* TAA04, *L. curvatus* TDM40, and *L. curvatus* TDB21 demonstrated superior tolerance, whereas at 45°C, *L. mesenteroides* TDB22, *P. pentosaceus* TAA01, and *L. mesenteroides* TDB19 yielded the highest GIs (Fig. 4). The strains in this study showed a higher average GI (89.32 ± 16.90%) at 15°C compared to the 83.08 ± 7.22% reported by Speranza et al. (2017). At 45°C, however, the strains exhibited a lower GI of 28.99 ± 16.03%, in contrast to the higher GI reported in the same study (Speranza et al., 2017). The high tolerance of *L. curvatus* strains to low temperatures suggests that they may have psychrotrophic traits, including the ability to produce cold-shock proteins. These proteins assist the bacteria in managing stress caused by sudden drops in temperature, enabling them to make transient metabolic adaptations (Capozzi et al.,2011; Vermeiren et al., 2004).

The temperature tolerance profiles of the LAB strains highlight their potential for industrial fermentation under varying thermal conditions. *L. curvatus* strains (TAA04, TDM40, and TDB21) show strong tolerance at 15°C, making them suitable for low-temperature fermentation. Previous study has reported that aroma intensity, overall balance, and product stability are improved in foods fermented at lower temperatures (Massera et al., 2021). In contrast, strains like *L. mesenteroides* TDB22, *P. pentosaceus* TAA01, and *L. mesenteroides* TDB19, which relatively tolerate temperatures at 37°C and above, can support fermentation at higher temperatures, potentially reducing spoilage by inhibiting competing microorganisms (Bevilacqua et al., 2010). Therefore, selecting LAB strains based on their thermal tolerance can enhance fermentation efficiency.

**Acid tolerance:** Apart from osmotic pressure and temperature variation, acidity is a critical variable influencing the performance of LAB strains in *Tella* fermentation (De Angelis & Gobbetti, 2004). Since the pH value of *Tella* ranges from 4.0 to 5.0 (Lee et al., 2015; Tekle et al., 2019), pH values of 3.5, 4.0, and 4.5 were selected to measure the acid tolerance ability of the strains. The strains’ growth properties were significantly affected by acidic conditions at all tested pH values (Fig. 4), displaying a wide variation in tolerance levels. At pH 3.5, the strains yielded GI values ranging from 15.50 ± 0.08% to 72.02 ± 0.03%, with an average GI of 40.98 ± 21.84%. At pH 4.0, more than 70% of the strains exhibited a GI > 50%. Among the strains incubated at pH 4.0, *L. plantarum* TDM41 yielded the highest acid tolerance, with a GI value of 89.29 ± 0.04%, followed by *P. pentosaceus* TAA01 and *L. mesenteroides* TDB19, with GI values of 62.33 ± 0.07% and 61.64 ± 0.14%, respectively. At pH 4.5, the strains exhibited higher GI values, ranging from 68.22 ± 0.08% to 95.24 ± 0.61%. Notably, across all pH levels, *L. plantarum* TDM41 consistently demonstrated the highest GI values, whereas *L. curvatus* TDM40 consistently displayed the lowest GI values. Previous studies have reported similar results regarding the acid tolerance properties of LAB (Bevilacqua et al., 2010; Goswami et al., 2017; Van De Guchte et al., 2002). According to Bevilacqua et al. (2010), the GI of *L. plantarum* strains incubated at pH 4.0 and 37°C ranged from 72.00% to 112.00%. In this study, *L. plantarum* TDM41 yielded an 89.29% GI at pH 4.0 when incubated at 30°C. With the pH of fermented cereals like *Tella* typically ranging from 3.5 to 4.5 (Alemayehu, 2018; Lee et al., 2015; Montemurro et al., 2020), three strains of *L. plantarum* TDM41, *P. pentosaceus* TAA01, and *L. mesenteroides* TDB19 demonstrated better acid tolerance, making them potential candidates for acidic food fermentations. Among them, *L. plantarum* TDM41 stood out with consistently higher GI values across all tested pH levels, highlighting its superior adaptability and promising potential to enhance the stability and quality of *Tella* and other acidic fermented foods.

During fermentation, LAB produce acidic end products, primarily lactic acid, which accumulate in the extracellular environment. As lactic acid concentration increases, it triggers resistance mechanisms, including the maintenance of pH homeostasis, cell membrane integrity, metabolic regulation, and macromolecule repair (Guan & Liu, 2020). These mechanisms help mitigate the damage caused by acidic environments, enabling LAB to survive and thrive under such conditions. However, when acid concentration exceeds a certain threshold, intracellular pH declines, disrupting pH homeostasis and potentially causing protein and DNA damage, which can lead to cell death (Wu et al., 2012). Thus, strains such as *L. plantarum* TDM41, *P. pentosaceus* TAA01, and *L. mesenteroides* TDB19, with strong acid tolerance, are crucial for supporting efficient fermentation at acidic conditions and ensuring strain viability under gastrointestinal conditions, making them beneficial for probiotic applications as well (Bevilacqua et al., 2010).

**Fig. 5.**
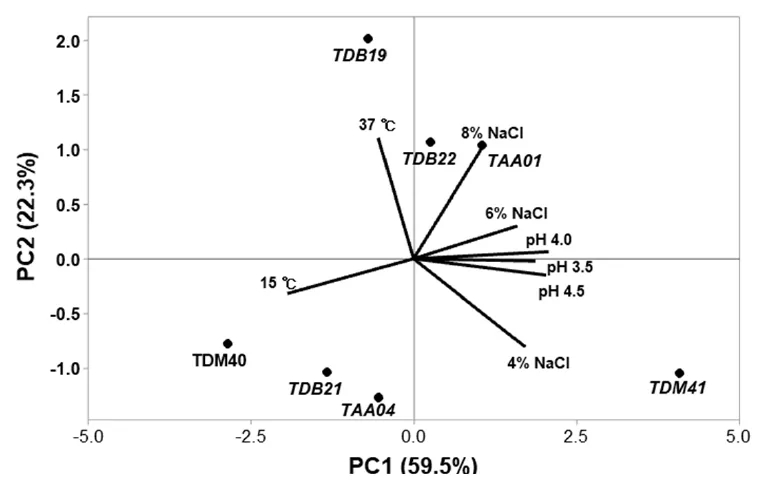
Principal component analysis (PCA) biplot based on the stress tolerance properties (growth index at 4% NaCl, 6% NaCl, 8% NaCl, 15°C, 37°C, pH 3.5, pH 4.0, and pH 4.5 in MRS broth) of seven LAB strains: *P. pentosaceus* TAA01, *L. curvatus* TAA04, *L. mesenteroides* TDB19, *L. curvatus* TDB21, *L. mesenteroides* TDB22, *L. curvatus* TDM40, and *L. plantarum* TDM41.

**Selection of the best stress-tolerant strains:** The best stress-tolerant strains were selected using a multivariate technique, namely, PCA (Fig. 5.). PCA of stress tolerance properties was performed on eight parameters: tolerance to salt (growth at 4, 6, and 8% NaCl over 48 h at 30°C), tolerance to temperature (growth at 15 and 37°C over 48 h, and tolerance to acidic pH (growth at 3.5, 4.0, and 4.5 over 48 h). The similarity map defined by the first two principal components (PC1 and PC2) accounted for 81.80% of the total variance. Fig. 5 presents the variables and observation projections in the PC1 and PC2 space. PC1 and PC2 accounted for 59.50% and 22.30% of the total variance, respectively. An analysis of the contribution of variables to the principal components revealed that PC1 was mainly related to growth at 6% NaCl, 15℃, pH 3.5, pH 4.0, and pH 4.5, while PC2 was linked to growth at 37°C, 4% NaCl, and 8% NaCl. Strain dispersion over the four quadrants evidenced wide variability in the strains’ stress tolerance properties. Notably, all three *L. curvatus* strains (TDM40, TDB21, and TAA04) clustered in quadrant three, while *L. mesenteroides* strains TDB19 and TDB22 were located near each other in quadrants one and two. *L. plantarum* TDM41 appeared alone in quadrant four, and *P. pentosaceus* TAA01 was positioned in quadrant two. These observations suggest that the stress tolerance properties of these strains are closely linked to their genetic backgrounds (Sánchez García et al., 2007). The biplot allowed us to distinguish three LAB strains: *L. plantarum* TDM41, *P. pentosaceus* TAA01, and *L. mesenteroides* TDB22, which present better stress tolerance properties.

### Acidification ability of LAB strains

**Fig. 6.**
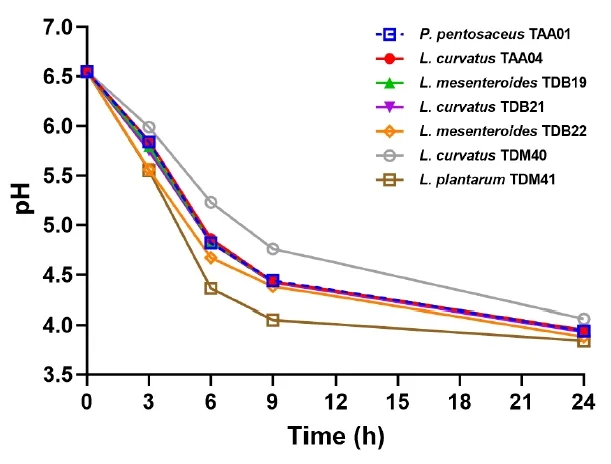
Rate of acidification of sterile *Tella* substrate extract fermented with LAB strains after 0, 3, 6, 9, and 24 h of fermentation. Values are expressed as the mean ± SD (n=3).

The pH value of *Tella* usually ranges from 4.00 to 5.00 (Lee et al., 2015; Tekle et al., 2019). Another researcher reported a lower pH value of 3.28 for *Tella* (Alemayehu, 2018). Spontaneous cereal-based alcoholic fermentations are typically associations of LAB and yeast, as yeast growth is favored by acidification (Blandino et al., 2003; Nout & Sarkar, 1999; Sáez et al., 2017). In addition, a low pH is important for preventing the growth of adventitious microflora (Sáez et al., 2017). Consequently, acidification ability is among the crucial technological properties governing LAB selection for *Tella* and other LAB and yeast-associated fermentations (Sáez et al., 2017). In this study, the initial pH of STSE was adjusted to 6.5 at the onset of incubation. Due to the acidification performance of the strains, the pH declined below pH 4.0 after 24 h of fermentation, except for *L. curvatus* TDM40 (Fig. 6). The reduction in pH observed during the fermentation period is mainly attributed to acid production, with lactic acid being the predominant type (Nuryana et al., 2019). The strains’ rates of acidification ranged from 0.56 ± 0.04 to 1.01 ± 0.06, 1.32 ± 0.04 to 2.19 ± 0.04, 1.79 ± 0.04 to 2.5 ± 0.04, and 2.49 ± 0.04 to 2.71 ± 0.04) after incubation times of 3, 6, 9, and 24 h, respectively (Fig. 6). Throughout the fermentation period, *L. plantarum* TDM41 exhibited the highest rate of acidification, followed by *L. mesenteroides* TDB22 and *P. pentosaceus* TAA01. In contrast, *L. curvatus* TDM40 yielded the lowest rate of acidification. The acidification rates observed in the present study are notably higher than those reported by Sawadogo‐Lingani et al. (2008), who documented acidification rates of *L. fermentum* strains in autoclaved sorghum malt broth, with rates ranging from 0.03 ± 0.01 to 0.67 ± 0.01, 0.1 ± 0.01 to 1.38 ± 0.02, and 0.38 ± 0.01 to 1.67 ± 0.01 after 3, 6, and 9 h of incubation, respectively. Furthermore, nearly all strains in this study reduced the pH of STSE to below 4.00, demonstrating a more pronounced acidification compared to the findings of Sáez et al. (2017), who observed that various LAB strains, including *Enterococcus durans*, *L. rhamnosus*, *Lactococcus garvieae*, and *Weissella cibaria*, lowered the pH of abulia flour extract from 6.50 to a range of 4.07–4.96 after 24 h of incubation. Additionally, the superior acidification performance of *L. plantarum* strains over *L. curvatus* and *L. mesenteroides* observed in this study is consistent with the findings of Manini et al. (2016) in wheat bran dough fermentation. Similarly, *P. pentosaceus* and *L. mesenteroides* strains exhibited high acidification activity, lowering the fermentation substrate pH to 3.50 in sourdough, sauerkraut, and skim milk fermentation (Guetarni, 2024; Montemurro et al., 2020; Xiong et al., 2014), which aligns with our study. However, the acidification observed in these studies was more intense compared to the results of the present study. The pH reduction occurs as LAB strains utilize available sugars and convert them into organic acids (Celik et al., 2010; Chen et al., 2019; Zvauya et al., 1997). In traditional fermentation processes like *Tella*, the fermentation typically spans several days. A rapid decrease in pH during the initial step of *Tella* fermentation is requisite to accelerating the fermentation process. Fast-acidifying strains, such as *L. plantarum* TDM41, may be suitable candidates in most fermentation processes as primary starter organisms, whereas slow-acidifying strains can be used as adjunct cultures depending on their additional contribution to product quality and safety (Salvucci et al., 2016). In addition, the acidification ability of strains potentially contributes to the flavor, texture, and nutritional characteristics of products (Gänzle, 2014).

### Organic acid production profile of LAB strains

Organic acids play an important role in maintaining the nutritional value, sensory quality, and shelf life of foods (Ferreira et al., 2022; Wang et al., 2023). The production of selected organic acids by each LAB strain in STSE is presented in Table 2. Acetic acid and succinic acid were not detected in the STSE. Lactic acid was more abundantly produced than other organic acids, with *L. curvatus* TAAO4, *L. mesenteroides* TDB19, *L. curvatus* TDB21, and *L. curvatus* TDM40 being the highest producers (range: 2.12–2.15 g/L). *L. curvatus* TAAO4, *L. mesenteroides* TDB19, *L. curvatus* TDB21, and *L. curvatus* TDM40 also displayed a higher production of malic acid, citric acid, acetic acid, and succinic acid than the other three strains. Among the LAB strains, *L. curvatus* TDM40 exhibited the highest production of malic acid, citric acid, and acetic acid, with values of 2.22, 1.47, and 0.26 g/L, respectively. All LAB strains generally demonstrated a lower production of succinic acid, ranging from 0.05 to 0.14 g/L.

**Fig. 7.**
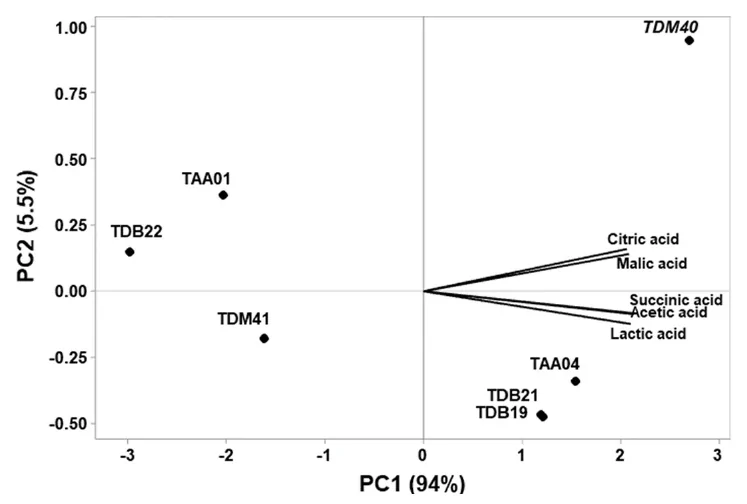
Principal component analysis (PCA) biplot of organic acids obtained via fermentation of sterile *Tella* substrate extract with seven LAB strains: *P. pentosaceus* TAA01, *L. curvatus* TAA04, *L. mesenteroides* TDB19, *L. curvatus* TDB21, *L. mesenteroides* TDB22, *L. curvatus* TDM40, and *L. plantarum* TDM41. Organic acid values are presented in Table 2.

To further elucidate and visualize the correlation between LAB strains and organic acids, PCA (Fig. 7) was conducted using organic acid data from Table 2. The first two principal components, PC1 and PC2, accounted for 94.0% and 5.5% of the total variance, respectively. The resulting biplot positioned the LAB strains according to their organic acid production capabilities across four quadrants, indicating distinct profiles. LAB strains in quadrants II and IV exhibited a stronger correlation with the production of all organic acids compared with those in quadrants I and III. Notably, *L. curvatus* TDM40 was positioned in the far-right corner, highlighting it as an outstanding producer of organic acids.

The unique taste of traditionally produced *Tella*, with its balance of sweetness, bitterness, and sourness, is primarily shaped by spontaneous fermentation, which involves a diverse microbial community (Tekle et al., 2019; Yohannes et al., 2013). The variety of microbes in this process plays a crucial role in developing the complex flavor, aroma, and texture of *Tella*. While controlled fermentation using one or a few specific strains can improve control and predictability, it may limit the natural complexity of these sensory attributes. Therefore, incorporating a mixed-culture approach, alongside carefully selected LAB strains, can enhance fermentation efficiency and help maintain *Tella*’s distinctive sensory qualities (Smid & Lacroix, 2013).

### Selection of potential LAB candidates for *Tella* starter culture

To select the best starter culture candidates for *Tella* fermentation, evaluating a comprehensive set of technological properties is essential, as relying on a few criteria is insufficient. Most strains demonstrated similar carbohydrate utilization, with *L. plantarum* TDM41 displaying exceptional performance. Additionally, the strains exhibited similar growth characteristics. In terms of stress tolerance and acidification potential, *L. plantarum* TDM41, *P. pentosaceus* TAA01, and *L. mesenteroides* TDB22 performed best. However, the strains that excelled in organic acid production were *L. curvatus* TAAO4, *L. curvatus* TDB21, and *L. curvatus* TDM40, which enhanced the flavor complexity of *Tella*. Among the top-performing strains in stress tolerance and acidification potential, *L. plantarum* TDM41 generally demonstrated the highest production of organic acids, such as lactic acid, acetic acid, and succinic acid. *P. pentosaceus* TAA01 displayed a higher production of citric acid and similar malic acid than *L. plantarum* TDM41. Conversely, *L. mesenteroides* TDB22 consistently produced the lowest levels of all measured organic acids.

The aim of selecting a starter culture for *Tella* fermentation is to enhance its functionality while ensuring a standard and controlled efficient process that elicits its probiotic benefits (Yehuala et al., 2024). The major challenge in the development of probiotic alcoholic beverages is maintaining probiotic viability during processing and storage (Chan et al., 2019). Starters in these beverages are stressed by factors such as ethanol content, hope, heat, and low pH (Berhanu, 2014; dos Santos et al., 2023; Hinojosa-Avila et al., 2023). According to Vinicius De Melo Pereira et al. (2020), essential criteria for selecting starter cultures include tolerance to heat, alcohol, and organic acids as well as the ability to produce beneficial metabolites. Based on these criteria, *L. plantarum* TDM41 and *P. pentosaceus* are promising candidates for controlled *Tella* fermentation owing to their superior technological properties. These strains have also demonstrated potential in other technological applications (Manini et al., 2016). Additionally, incorporating *L. curvatus* strains, noted for their production of significant organic acids, may enhance the flavor profile of the final product in multi-culture systems.

## Conclusion

Selecting LAB starters satisfying the demands of the modern food industry requires thorough research into their properties. Key technological properties, such as carbohydrate fermentation potential, growth characteristics, stress tolerance properties, acidification abilities, and organic acid production profiles are imperative. The LAB strains studied demonstrated significant variability of these technological properties. When no single strain possesses all beneficial properties, employing multi-culture systems may be necessary. *L. plantarum* TDM41 and *P. pentosaceus* TAA01 have been proposed as the most promising candidates for *Tella* and other industrial starter cultures. However, further research is warranted, especially on their interaction with yeast cells in co-culture and multi-culture systems as well as large scale settings, to achieve the desired product qualities.

## Figures and Tables

**Table 1.** Carbohydrate utilization by LAB strains

LAB strains	Carbohydrate utilization					
	Glucose	Sucrose	Maltose	Fructose	Galactose	Sorbitol
*P. pentosaceus* TAA01	++	++	++	++	++	–
*L. curvatus* TAA04	++	++	++	++	++	–
*L. mesenteroides* TDB19	++	++	++	++	++	–
*L. curvatus* TDB21	++	++	++	++	++	+
*L. mesenteroides* TDB22	++	++	++	++	++	–
*L. curvatus* TDM40	++	++	++	++	++	–
*L. plantarum* TDM41	+++	+++	+++	+++	+++	+

+++, high utilization (ΔpH ≥ 3.5); ++, medium utilization (2.0 ≤ ΔpH < 3.5); +, low utilization (0.5 ≤ ΔpH < 2.0); –, no utilization (ΔpH < 0.5) after 48-h incubation at 30°C

**Table 2.** Concentrations of organic acids in *Tella* substrate fermented with different LAB strains

Samples	Organic acid concentrations (g/L)				
	Lactic acid	Acetic acid	Citric acid	Malic acid	Succinic acid
T1	0.02 ± 0.00^e^	ND	0.03 ± 0.00^f^	0.02 ± 0.00^e^	ND
T2	1.09 ± 0.04^c^	0.08 ± 0.00^b^	0.72 ± 0.04^c^	0.81 ± 0.05^c^	0.07 ± 0.00^a^
T3	2.12 ± 0.11^a^	0.25 ± 0.01^a^	1.07 ± 0.05^b^	1.53 ± 0.08^b^	0.14 ± 0.01^a^
T4	2.13 ± 0.11^a^	0.24 ± 0.01^a^	0.99 ± 0.05^b^	1.43 ± 0.07^b^	0.13 ± 0.01^a^
T5	2.15 ± 0.11^a^	0.24 ± 0.01^a^	0.99 ± 0.05^b^	1.44 ± 0.07^b^	0.13 ± 0.01^a^
T6	0.85 ± 0.04^d^	0.07 ± 0.00^b^	0.48 ± 0.02^e^	0.62 ± 0.03^d^	0.05 ± 0.00^a^
T7	2.15 ± 0.11^a^	0.26 ± 0.02^a^	1.47 ± 0.07^a^	2.22 ± 0.13^a^	0.14 ± 0.01^a^
T8	1.38 ± 0.06^b^	0.11 ± 0.01^b^	0.65 ± 0.03^d^	0.81 ± 0.04^c^	0.08 ± 0.00^a^

T1, sterile *Tella* substrate extract (STSE); T2−T8, STSE samples fermented with respective strains; ND, not detected. Values are expressed as the mean of triplicate experiments ± SD. Different lowercase letters in a column indicate statistically significant differences (P < 0.05) among the organic acid contents of the STSE samples fermented with different strains

## Data Availability

All the data related to this study has been presented in the article.
